# ‘Hit and run’ serial femtosecond crystallography of a membrane kinase in the lipid cubic phase

**DOI:** 10.1098/rstb.2013.0621

**Published:** 2014-07-17

**Authors:** Martin Caffrey, Dianfan Li, Nicole Howe, Syed T. A. Shah

**Affiliations:** Membrane Structural and Functional Biology Group, School of Medicine and School of Biochemistry and Immunology, Trinity College Dublin, Dublin 2, Republic of Ireland

**Keywords:** crystal structure, enzyme, membrane protein, mesophase, monoacylglycerol, X-ray free-electron laser

## Abstract

The lipid-based bicontinuous cubic mesophase is a nanoporous membrane mimetic with applications in areas that include medicine, personal care products, foods and the basic sciences. An application of particular note concerns it use as a medium in which to grow crystals of membrane proteins for structure determination by X-ray crystallography. At least two variations of the mesophase exist. One is the highly viscous cubic phase, which has well developed long-range order. The other so-called sponge phase is considerably more fluid and lacks long-range order. The sponge phase has recently been shown to be a convenient vehicle for delivering microcrystals of membrane proteins to an X-ray free-electron laser beam for serial femtosecond crystallography (SFX). Unfortunately, the sponge phase approach calls for large amounts of protein that are not always available in the case of membrane proteins. The cubic phase offers the advantage of requiring significantly less protein for SFX but comes with its own challenges. Here, we describe the physico-chemical bases for these challenges, solutions to them and prospects for future uses of lipidic mesophases in the SFX arena.

## 1. Introduction

Serial femtosecond X-ray crystallography (SFX) is a relatively new method for collecting crystallographic information on small crystals fed continuously into a free-electron laser (FEL) beam composed of high-fluence X-ray bunches mere femtoseconds long [1,2]. Each encounter between an X-ray bunch and a microcrystal (*hit*) ideally gives rise to a single, still diffraction pattern with greater than 10 measurable reflections. As the crystals are randomly oriented, collecting patterns on enough crystals (thousands typically) produces a complete dataset of high redundancy for structure determination, to date by molecular replacement [3–7]. Data are collected in an evacuated interaction sample chamber operated at 20°C. Despite the intensity of the X-ray bunch (typically 10^12^ photons/bunch), each is of such short duration that insufficient time is available for the changes associated with radiation damage to progress sufficiently before the diffracted X-rays have departed (*run*) with their structural manifest to be recorded. We refer to this as ‘*hit and run’* SFX.

Until the feasibility study described in this work was carried out, a fluid medium had been used to ferry crystals of membrane proteins for SFX [3,7]. The process involved voluminous flow rates. Because productive interactions between X-rays and crystals in the flowing stream were so infrequent, vast amounts of valuable membrane protein were required for data collection and most of the protein went to waste. Typically, only 1 in 25 000 crystals produced a useful diffraction pattern. Thus, for example, when photosystem I (PSI) crystals were used dispersed in a liquid jet, data collection required 10 mg of protein [3]. By contrast, when photosynthetic reaction centre crystals were delivered dispersed in the lipidic sponge phase, 3 mg of protein were needed [7]. The idea was subsequently mooted that using the highly viscous lipid cubic phase (LCP), in which membrane proteins can be grown by the *in meso* method, might provide an alternative transport medium for SFX. As a result of being viscous, flow rate would be reduced dramatically. And if high enough crystal densities in the LCP could be achieved, the rate of delivery of crystals and X-rays to the interaction region could be matched for a most efficacious use of both. The method is hereafter referred to as LCP-SFX.

LCP-SFX is appealing as a method, because it offers the prospect of obviating some of the issues that arise with *in meso*-based structure determination using synchrotron X-radiation. With the *in meso* method, crystals are typically grown in a sealed glass sandwich plate. Harvesting crystals is a somewhat cumbersome process that can lead to substantial loss of crystals and to degradation in diffraction quality. Data collection at a synchrotron source is typically done at 100 K. Such a frigid temperature can stabilize conformational substates, particularly in the protein's side chains, that are not physiologically relevant and that are possibly misleading as far as functional interpretation is concerned [8]. Radiation damage is also a major concern with synchrotron radiation sources where residues such as aspartate and glutamate are particularly prone to undergo decarboxylation [9]. Damage can be mitigated to a degree with large crystals, beam attenuation and data collection at cryo-temperatures often requiring many tens of crystals. In this context, then, LCP-SFX was attractive in that it offered what amounts to *in situ* data collection with micro- or nanometre-sized crystals at or close to the more physiologically relevant 20°C and the prospect of outrunning radiation damage.

Diacylglycerol kinase (DgkA) was the membrane protein with which this study was performed. DgkA is a small, 121-residue homo-trimer with three active sites of the shared sites type. It catalyses the ATP-dependent phosphorylation of diacylglycerol to form phosphatidic acid. Phosphatidate is subsequently used to shuttle water-soluble components for lipopolysaccharide and membrane-derived oligosaccharide synthesis in the cell envelope of Gram-negative bacteria [10]. The protein had been previously crystallized by the *in meso* method and its structure was determined to 2.05 Å using a thermostabilized and fully functional mutant [11]. Structure determination required extensive crystallization condition screening and optimization, much of which involved host lipid and temperature screening [12]. The final structure was solved using crystals grown in 7.8 monoacylglycerol (MAG) as the host lipid at 4°C. Diffraction data were collected using synchrotron radiation at 100 K. The structure revealed a number of aspartates and glutamates in the putative active site that had previously been identified by mutagenesis studies as key players in the catalytic mechanism of this diminutive kinase [13,14]. SFX, with its ability to outrun radiation damage, was considered attractive as an alternative method for structure determination where the pristine, undamaged state might be revealed.

**Figure 1.**
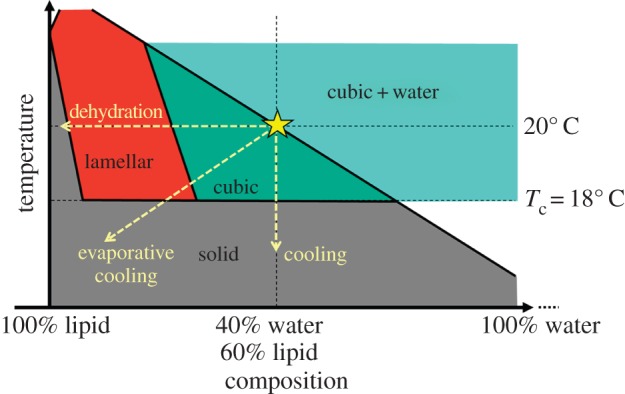
Schematic of the equilibrium temperature–composition phase diagram for the monoolein (9.9 MAG)–water system in the vicinity of 20°C. The different phases are shown as coloured zones and labelled accordingly. The cubic mesophase is extruded into the evacuated sample chamber for SFX under conditions indicated by the yellow star at 20°C and approximately 40% aqueous medium. Possible trajectories through the phase diagram taken upon dehydration, cooling and evaporative cooling are indicated by dashed arrows. The 20°C isotherm is identified by a horizontal dashed line. The liquid crystal-to-solid (*L*_c_) transition is identified by the horizontal dashed line at 18°C. This schematic is based on the equilibrium phase diagram for 9.9 MAG reported in [15]. (Online version in colour.)

The lipid cubic phase takes centre stage in this work. It serves as the medium in which crystallization occurs and is, in turn, used to port those same crystals into the X-ray free-electron laser (XFEL) beam for SFX. As a lyotropic liquid crystal, it is formed most simply when monoolein and water, in approximately equal parts, are mixed together at 20°C (figure 1). By spontaneous self-assembly, the mesophase forms in a process driven primarily by the hydrophobic effect. As with any state of matter, mesophase behaviour is dictated by the Gibbs’ phase rule and is conveniently and concisely summarized in the form of a temperature–composition phase diagram. The equilibrium phase diagram for the monoolein–water system has been mapped out based on small- and wide-angle X-ray scattering measurements [15,16]. It shows that the cubic phase gives way to a solid, the lamellar crystalline or *L*_c_ phase, below about 18°C. The cubic phase consists of a single, continuous highly curved and branched lipid bilayer on either side of which is a bathing aqueous channel. These two continuous channels interpenetrate but never contact one another directly because a lipid bilayer separates them. For use in *in meso* crystallogenesis, the mesophase is prepared typically by combining the host lipid with an aqueous solution of a pure membrane protein solubilized in detergent [17]. The most commonly used host lipids are *cis*-monoenoic monoacylglycerols (MAGs) with acyl chains 14–18 carbon atoms long [12,18]. A lipid synthesis programme in the Membrane Structural and Functional Biology Group's laboratory provides these MAGs in support of the *in meso* method of crystallization [19]. Given their success, several are now commercially available.

A noted feature of the cubic phase is its viscosity. The older phase science literature referred to it as the viscous isotropic or VI phase, reflecting its ‘challenging’ rheological and non-birefringent optical properties. Viscosity has been highlighted by some as an undesirable property of the cubic phase as a medium with which to perform crystallization. However, there are several applications where its sticky and viscous nature offer distinct advantages. As a medium for porting crystals of membrane proteins into the XFEL beam for SFX is one such example, as demonstrated convincingly below. Despite its viscosity and challenging handling properties, a robot was built that enables the setting up of *in meso* crystallization trials in high-throughput fashion using miniscule quantities of mesophase and protein [20]. Most trials are set up now using anywhere from 20 to 50 nl of mesophase corresponding to approximately 5–20 ng protein/well. Given the success of this robot, variations on the original design are available commercially [21].

It is the bicontinuous nature of the LCP that is at the heart of *in meso* crystallogenesis. Our working hypothesis for how crystallization takes place begins with the target protein reconstituted into and uniformly distributed throughout the continuous, bilayered membrane that permeates the mesophase. Components of the precipitant stabilize a transition locally to the lamellar phase into which proteins diffuse to preferentially partition, concentrate and subsequently nucleate giving rise to macroscopic crystals [22]. The latter are noted for generally tending to be small, but of high diffraction quality, and major effort is usually required to optimize conditions that produce crystals large enough for synchrotron radiation-based data collection.

Despite the challenges of the method, it has been used to generate crystal structures of a number of different membrane proteins and complexes [17]. The most notable, of late, was the β_2_ adrenergic receptor–Gs protein complex that was the subject of the 2012 Nobel Prize in Chemistry [23]. To date, almost 170 record entries in the Protein Data Bank (PDB, www.pdb.org) are attributed to the *in meso* method. This corresponds to about 11% of all membrane protein structure records in the PDB. Attesting to the growing interest in the method, almost 60 new *in meso* records have been added to the PDB since the beginning of 2012.

## 2. Technical challenges

With a view to implementing LCP-SFX, three major challenges of a technical nature were identified. These included vacuum incompatibility of the monoolein-based LCP, the need to scale up from nanolitre to microlitre volumes of crystal-laden mesophase, and the provision of an injector that could extrude the highly viscous mesophase in the form of a micrometre-diameter, continuous bolus into the XFEL beam. The latter LCP injector was developed as part of a collaboration between Uwe Weirstall's group at Arizona State University and Vadim Cherezov's group at The Scripps Research Institute [24], and will only briefly be referred to at the end of this report.

### 2.1. Host lipid, vacuum compatibility

**Figure 2.**
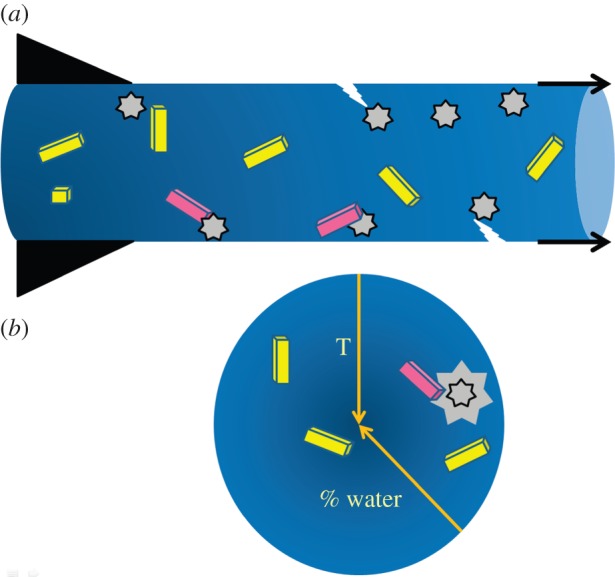
Cartoon representation of the crystal-laden mesophase bolus as it is extruded through the nozzle (black triangles) of the LCP injector into the evacuated sample chamber at 20°C for serial femtosecond crystallographic measurements with an X-ray free-electron laser (XFEL). (*a*) Side view of the bolus where the gradient in colour from left to right corresponds to the gradient in temperature and composition along the length of the bolus induced by evaporative cooling. (*b*) End on view of the bolus where the gradient in colour corresponds to the gradient in temperature and composition (arrows) along the radius of the cylindrical bolus induced by evaporative cooling. Pristine, undamaged membrane protein crystals are coloured yellow and are shown dispersed in a blue cubic mesophase. Stars correspond to sites where the mesophase has transformed from the cubic to the solid *L*_c_ phase that may damage the crystals (red) and introduce defects (lightning bolt) in the bolus thereby affecting flow. The star in (*b*) with the enlarged grey background is drawn to suggest local heating due to the heat of fusion associated with the solidification reaction that may damage dispersed crystals nearby. (Online version in colour.)

The first technical challenge referred to above relates to the phase behaviour of the medium in which crystals are grown and then ported into the XFEL beam for SFX. As noted, data are collected in an evacuated sample chamber at 20°C. The mesophase is extruded from the injector as a fully hydrated bolus in which microcrystals are dispersed. Immediately upon entering the chamber, volatiles (water in particular) will evaporate from the surface of the bolus and, by evaporative cooling, the sample temperature will drop (figure 2). Evaporation leads to increases in the concentration of all non-volatiles in the bolus. These include lipid, detergent, protein, protein crystals, and buffer and precipitant components. Accordingly, concentration gradients develop along the length and across the diameter of the cylindrically shaped mesophase bolus. The magnitude of the gradients depends on flow rate and distance along the bolus from the tip of the injector nozzle. Depending on the final concentrations reached, these assorted components can crystallize directly and/or destabilize the dispersing mesophase.

As noted, mesophase behaviour is dictated by temperature and composition [15,16] (figure 1). Evaporative cooling, brought about by loss of water, will induce sample cooling as well as an increase in lipid concentration. From a perusal of the equilibrium temperature–composition phase diagram for the monoolein/water system (figure 1), a reduction in water content together with a drop in temperature will give rise to a transition from the cubic mesophase to the solid *L*_c_ phase [15,16]. This change in phase can have detrimental consequences. These include a change in the rheological and flow characteristics of the extruded jet creating problems with sample positioning in the XFEL beam. Because the transition has a large associated heat of fusion, wherever crystallization occurs a local ‘hot’ spot will develop that, in turn, may impact negatively on jet flow characteristics and on membrane protein crystal quality. Furthermore, the *L*_c_ phase itself, as a solid, may damage the delicate membrane protein crystals dispersed in the bolus. Detrimental too is the background powder diffraction from the bolus medium which is strong and sharp both at low and wide angles in the case of the *L*_c_ phase. This is in contrast to the cubic phase which gives rise to relatively benign diffuse scattering at wide angles, although diffraction in the low-angle region can be strong and sharp. Background scatter from the *L*_c_ phase creates problems for the recovery of crystal diffraction data from recorded composite images. More importantly, it can damage the detector, and it is partly for this reason that the incident beam was attenuated some 20-fold during data collection in the current feasibility study.

It was considered important therefore to avoid the undesirable cubic-to-*L*_c_ transition due to evaporative cooling. An obvious way around this was to reduce the cubic-to-*L*_c_ transition temperature, *T*_c_, which could be achieved by using an alternative host MAG to monoolein (9.9 MAG) whose *T*_c_ is 18°C (figure 1). Separately, we had designed 7.9 MAG for *in meso* crystallization at low temperatures [25]. The *T*_c_ of 7.9 MAG under conditions of full hydration is about 6°C. This was deemed low enough for this application, and 7.9 MAG was chosen as the host lipid for use in the feasibility study with DgkA. It was subsequently shown to behave as expected and to prevent the formation of the *L*_c_ phase under conditions of SFX data collection.

As noted in §1, the 2.05 Å structure of DgkA was obtained after extensive crystallization screening and optimization [11,12]. Final crystals were generated in 7.8 MAG at 4°C. It was necessary therefore to rescreen and optimize in 7.9 MAG, and ideally this should be done at 20°C, the temperature at which SFX data were to be collected. Because a corresponding structure determined using synchrotron radiation was not available for crystals grown in 7.9 MAG, it was necessary to generate crystals for that purpose in 7.9 MAG and, again, ideally at 20°C. However, the crystal requirements for synchrotron radiation and for SFX data collection are entirely different. For the former, a few large single crystals suffice. For SFX, tens of microlitres of mesophase containing a high density of micrometre-sized crystals are needed.

Adjusting the concentration of 2-methyl-2,4-pentanediol (MPD) in the precipitant solution provided crystals in 7.9 MAG at 20°C that ranged from showers of microcrystals, as required for SFX, to isolated, relatively large single crystals suitable for synchrotron radiation data collection. The former were used successfully for SFX and provided a structure of DgkA to approximately 2.2 Å. The latter, however, diffracted at the synchrotron to no better than 6 Å. Additional rounds of optimization, performed at 4°C, resulted in large, single crystals that provided a synchrotron radiation structure at 2.2 Å. Structure details and comparisons will be reported separately.

### 2.2. Scaling up

**Figure 3.**
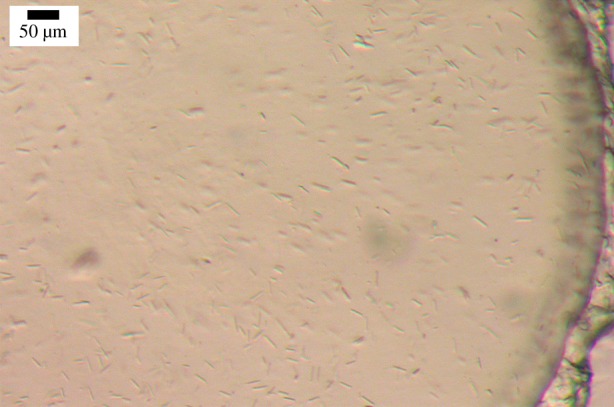
Microcrystals of DgkA grown in the cubic mesophase with 7.9 MAG as host lipid at 20°C in a 0.5 ml syringe. Details of sample preparation are described in [27]. (Online version in colour.)

The next technical challenge required increasing the scale of crystallogenesis. For SFX, it was anticipated that tens of microlitres of crystal-laden mesophase would be required to collect enough data for a structure solution. As noted, *in meso* crystallization screening is highly efficient and is performed typically on a 50 nl mesophase per well basis [17]. The challenge then was to scale up crystallogenesis by about a 1000-fold. Owing to the scarcity of the DgkA protein itself, as with most membrane proteins, extensive screening for optimum crystallization conditions is practically impossible on such a large scale. It was necessary therefore to identify conditions that produced a high density of microcrystals, first of all under standard conditions in glass sandwich plates at the 50 nl level, and to subsequently scale up with the expectation that these same conditions would translate when scaled up by a factor of 1000. However, for the conditions to translate it was necessary to perform the large volume crystallogenesis while maintaining, as much as possible, the same geometrical relationships between mesophase bolus and precipitant solution as prevailed during nanolitre volume crystal growth. The geometry in question relates to the shape and size (diameter in particular) of the bolus in contact with the bathing precipitant solution. As the progress of *in meso* crystallization has been reasoned to depend on such factors [22], every effort was made to replicate those conditions for large-scale microcrystal production. This was realized by carrying out crystal growth in a bolus of protein-laden mesophase approximately 15 cm long and 0.4 mm in diameter located towards the centre of the barrel of a 0.5 ml Hamilton syringe containing precipitant solution. The composition of the precipitant solution had been identified to generate the desired, high density of microcrystals in standard nanolitre-scale glass sandwich plate screening. Upon incubation at 20°C for 5 days, needle-shaped microcrystals up to 30 μm long were obtained in high density, as expected. Most of the excess bathing precipitant solution was removed mechanically with the aid of an empty syringe and a narrow bore syringe coupler [26]. The last vestiges of residual excess precipitant were incorporated lyotropically by combining the opaque dispersion with a small volume of 7.9 MAG. This procedure generated the required bulk volume of optically clear cubic mesophase in which microcrystals (figure 3) were uniformly dispersed and ready for SFX measurements *in vacuo* at 20°C.

### 2.3. Lipid cubic phase injector

The third challenge associated with realizing LCP-SFX was the development, building and implementation of an injector capable of delivering at a fixed rate the highly viscous, crystal-laden mesophase as a uniform, continuous micrometre-diameter extruded bolus to the interaction region in the evacuated sample chamber at 20°C. This remarkable feat of engineering was realized, and the LCP injector has been described separately [24]. In operation, it involves the extrusion of mesophase from a 20 μl reservoir through a 6 cm-long glass capillary with an internal diameter of 20–50 μm. The tapered end of the capillary extends beyond the tip of a specially designed gas virtual nozzle which provides a co-flowing stream of gas for reliable, coaxial mesophase extrusion. Pressure, generated by a HPLC pump, is transmitted through water to the mesophase with a pair of Teflon beads separating and providing a water-tight seal between the two media.

## 3. Results

Having overcome all three technical challenges, the stage was set to proceed with the feasibility study. This was conducted using the Coherent X-ray Imaging (CXI) instrument at the Linac Coherent Light Source (LCLS) over the course of seven 12-h data collection shifts assigned to this DgkA and to several other membrane protein target projects in March 2013. The CXI operating conditions included: energy, 9.5 keV; wavelength, 1.3 Å; fluence, 10^12^ photons/bunch; average pulse energy at the sample, 0.05 mJ; bunch delivery rate, 120 Hz; bunch length, 50 fs; bunch diameter, 1.5 μm; attenuation, 3–6% of full beam. The injector was operated at an effective pressure up to 10 000 psi and a constant volumetric flow rate of 170 nl min^−1^ corresponding to a linear flow rate of 1.4 mm s^−1^, and the extruded bolus diameter was approximately 50 μm as defined by the 50 μm internal diameter of the nozzle capillary. The X-ray beam intersected the mesophase bolus approximately 100 μm from the tip of the capillary extending from the injector nozzle. The evacuated sample chamber was operated at 10^−4^ torr and 20°C. Diffraction data were collected on the Cornell-SLAC Pixel Array Detector (CSPAD) at a sample-to-detector distance of 122 cm. Images and diffraction data were analysed and processed following published procedures [28–30].

The data required to solve a structure of DgkA by molecular replacement were collected using approximately 4 h of beam time, 42 μl mesophase and 200 μg protein. Data collection was greatly facilitated by the high hit rate provided by the LCP jet. The SFX structure, at approximately 2.2 Å resolution, is very similar to the corresponding structures determined using synchrotron radiation at 100 K. A full analysis of the structure will be described separately. In addition, during the beam time assigned to this feasibility study structures were obtained for two liganded G protein-coupled receptors [27], and preliminary data were collected on several other integral membrane proteins and complexes. These will be reported separately.

## 4. Quo vadis?

The result of this spectacularly successful feasibility study can be summarized simply and succinctly as follows: LCP-SFX for membrane proteins works. Clearly, the method can and will be used with a host of other membrane proteins. However, this investigation was a feasibility study designed to determine whether LCP-SFX measurements were possible to begin with and, if so, to identify how future studies might be improved. Some thoughts along these lines are presented below.

A detector with wider dynamic range would certainly be of great benefit given that the current CSPAD required the beam to be attenuated by a factor of approximately 20 to prevent damage from strong and sharp reflections. The latter derive, in part, from membrane protein microcrystals themselves. But the biggest concern in this study was detector damage that might arise from the solid *L*_c_ phase induced to form by evaporative cooling of the host LCP. If the same experimental configuration, which includes an evacuated sample chamber, will be used for future LCP-SFX studies, then a better understanding is needed of the conditions that prevail in the extruded bolus under data collection conditions. These include knowing the temperature and composition along the length and across the diameter of the bolus and how these impact on phase behaviour of the various components in the porting medium. In the work reported here, the focus was on mesophase behaviour and how this was affected by changes in lipid hydration and temperature, with reference to the relevant temperature–composition phase diagram. The problem of converting to the solid *L*_c_ phase was averted by using a lipid, specifically 7.9 MAG, with a lower cubic-to-solid phase transition temperature [27]. There are other MAGs with low *T*_c_ values that can be used for this purpose. One, for which we have a detailed temperature–composition phase diagram (unpublished), is 9.7 MAG. An alternative approach, implemented successfully with the G protein-coupled receptors (GPCRs) used as part of this feasibility study, involved doping with the low *T*_c_ 7.9 MAG, mesophase prepared with a different hosting lipid or lipid mixture in which microcrystal had already grown and were dispersed. Monoolein containing 10 mol% cholesterol was the lipid mixture used in that application, and 7.9 MAG was added to the extent of 30 mol% post-crystal growth. The pre-existing crystals apparently did not suffer any deterioration in diffraction quality as a result of the doping exercise. However, for this to be a generally applicable procedure, checks on crystal quality must be performed and, as needed, doping protocols where damage is avoided or minimized must be developed. Should the post-crystal growth doping approach work with other hosting lipids and lipid mixtures, it will enormously expand the space available for crystallization screening in that screening will not be tied to a specific host lipid, as implemented with DgkA.

As noted, a synthesis programme in the Membrane Structural and Functional Biology Group provides rationally designed lipids for *in meso* crystallization and other applications in the membrane structural and functional biology field [19,25,31]. Separately, MAGs similar to those used in this study and other lipid types have been designed and synthesized for use in low-temperature crystallogenesis that may find application in future LCP-SFX studies. To do so, they must be shown to be effective hosting lipids for *in meso* crystallization and to form a crystal porting medium that is stable to evaporative cooling of the type that takes place during SFX data collection. Alternatively, they might be used to dope microcrystal-mesophase dispersions, thereby lowering the effective *T*_c_ and preventing evaporative cooling-driven solidification, as already demonstrated with GPCRs [27].

The cubic mesophase, with its rheological hallmark of viscosity, is integral to LCP-SFX. However, not all *in meso* screening efforts generate structure-quality crystals in the cubic phase. As often as not, the much more fluid, yet bicontinuous sponge phase is the medium from which final crystals emerge [17,32]. The sponge phase evolves from the cubic phase in the presence of certain precipitant components such as PEG 400, MPD and butanediol, and appears more prone to form with the shorter chain host MAGs [12]. It is characterized by significantly enlarged aqueous channels, long-range disorder, optical clarity, non-birefringence and fluidity. It is the latter property that makes it inefficient as a medium for porting microcrystals into the XFEL for SFX [7]. Of course, there are ways to reverse the process and to induce a sponge-to-cubic phase transition. These methods are used, for example, to facilitate crystal harvesting which, under certain conditions, is easier in the viscous cubic phase. The conversion is relatively easy to do when the ‘spongifying agent’ is an additive such as MPD where, typically, reducing the spongifier concentration in the sponge phase by dilution is sufficient to recover the cubic phase. Such an approach might be taken for LCP-SFX when final microcrystals only form in the sponge phase. Presumably, the conversion would be implemented immediately prior to running the SFX measurement and only in situations where it was shown that the process did not compromise crystal quality.

The *in meso* method is used primarily for crystallizing membrane proteins. However, it works also with soluble proteins. Lysozyme and thaumatin are cases in point [33,34]. It makes sense therefore to explore the utility of the LCP as a viscous, slow ‘flowing’ medium in which to port microcrystals of soluble proteins and complexes into the XFEL for efficient, high hit-rate SFX. Crystals can be grown *in situ* and used, essentially, as with membrane proteins. The alternative is to combine extant crystals with pre-formed mesophase to create a dispersion that can be loaded directly into the reservoir of the LCP injector for SFX measurement. In this latter case, the mesophase would best be prepared with the mother liquor in which the soluble protein crystals grew. As with membrane proteins, MAGs with different acyl chain characteristics and correspondingly different mesophase microstructures and rheologies should prove useful for generating and porting crystals of the widest possible range of soluble protein targets.
